# Developmental Histories Facilitating the Emergence of Creative Scientific Expertise: The Role of Developed Cognitive Talents, Education, and Social and Cultural Contexts

**DOI:** 10.3389/fpsyg.2021.716529

**Published:** 2021-09-03

**Authors:** Jonathan Wai, Matt I. Brown

**Affiliations:** ^1^Department of Education Reform and Department of Psychology, University of Arkansas, Fayetteville, AR, United States; ^2^Autism and Developmental Medicine Institute, Geisinger Health System, Lewisburg, PA, United States

**Keywords:** creativity, aptitudes and abilities, talent development, measurement, scientific expertise

## Abstract

Understanding how individual and contextual factors collectively contribute to the developmental histories that facilitate the emergence of creative expertise in science is improved by considering the contribution of the broad structure of developed cognitive abilities to creativity, prospective research on the high achieving or gifted students who may choose careers in and end up as creative scientists later in life, as well as retrospective studies of established creative scientists themselves and what their educational histories reveal. We first review and elaborate on these connections as documented in research which explore the development of talent, including cognitive mechanisms that include math and spatial reasoning and science related educational opportunities. We propose a research thought experiment that utilizes the multi-trait, multi-method matrix, and bifactor modeling to help understand the true overlap between measurement structures of cognitive and creative aptitudes. Then we explore the social and cultural contexts that may facilitate and/or hinder creative solutions in science through the lens of how these ecosystems influence talent development for gifted students and also the production of elite scientists. Based on this review, some policies will be suggested that may enhance the development of scientific creativity and broader societal innovation and expand the pipeline to include and fully develop the talents of disadvantaged students and provide nurturing environments to improve the likelihood of the emergence of scientific creative expertise.

## Introduction

The question of what are the most important factors in a developmental history, both individual and contextual, that go into the talent and educational life course of someone who ultimately makes a novel and creative contribution to science is a very old one. Some early studies of scientific expertise and creativity included those by Roe (1953a,b) who examined the *Making of a Scientist* by retrospectively comparing various characteristics and scientist's developmental trajectories across different fields. Super and Bachrach (1957) reviewed much of the literature to date on which factors were important to what we might term science, technology, engineering, and mathematics (STEM) achievement, and Tyler (1974) used an individual differences lens to examine achievement across a variety of domains, including in STEM areas. In referring to STEM fields in this paper we are largely referring to physical science, technology, engineering, and mathematics (pSTEM) fields (excluding life science and social science) (Ceci et al., 2014), though some of the findings likely do generalize to STEM conceptualized more broadly, thus we use the term STEM throughout the paper with full understanding that STEM is conceptualized differently by various researchers. Super and Bachrach (1957) specifically stressed the importance of math, verbal, and spatial reasoning in addition to various other attributes and contextual factors that mattered for STEM success, and West (1997) provided stories of scientific discovery where spatial reasoning or visualization was particularly important, which aligned with early empirical work by Smith (1964). Taken together, these investigations are consonant with research approaches which account for early attributes and developmental and contextual factors that collectively help understand STEM achievement and creativity (e.g., Feist, 2006; Lubinski and Benbow, 2006; Root-Bernstein and Pawelec, 2016; McCabe et al., 2020), as well as approaches to the development of talent and expertise (e.g., Subotnik et al., 2011; Hambrick et al., 2018).

Creativity in science is often recognized as a genuine scientific advance that is adequately supported by the evidence to date. Scientific advance, for example, can be quantified through combinations of publications, citations, awards, and impact (e.g., Simonton, 2004; Soler, 2007; Grosul and Feist, 2014; Anderson and Geist, 2017). Thus, this type of creativity is a constrained type of creativity which is novel only to the extent to which it holds up scientifically and with the test of time. For example, Einstein's theory of relativity and *Gedankenexperiments* might be characterized as uniquely creative in and of themselves, but they are a truly creative scientific advance only because they have been supported by experiment and the evidence. Therefore, creativity in many areas of science have a clear reality check that must happen for some advance to be considered creative at all. A more modern example is the development of mRNA technology and its clear usefulness grounded in the development of vaccines which now has widespread recognition primarily because of the COVID-19 pandemic (Kolata, 2021). For example, creativity researchers Plucker et al. (2004, p. 90) note “Our proposed definition is: Creativity is the interaction among *aptitude, process and environment* by which an individual or group produces a *perceptible product* that is both *novel and useful* as defined within a *social context*.” In the scientific social context, because true innovation in science is about making a real scientific advance that is grounded in scientific reality, it makes sense that developed cognitive aptitudes such as math, verbal or spatial reasoning would be important at least as a starting point for the opportunity to fully develop the expertise to eventually make a creative scientific discovery. Thus, developed cognitive aptitudes are important to consider in addition to creativity in helping understand how creative scientific expertise comes about in some cases but not in others.

## Roadmap of this Paper

We begin this paper by addressing the age-old question of whether creative aptitudes or abilities [e.g., divergent thinking (DT)] and cognitive aptitudes are related, and to what extent. We should make clear at the outset that creative aptitudes are different from creative production as defined by Plucker et al. (2004). We examine cognitive and creative aptitudes measure overlap as well as cognitive aptitudes and creative achievements overlap, including some consideration of long-term creative achievements. We then briefly discuss the threshold hypothesis, the idea that beyond a certain point more cognitive talent no longer matters for creativity or creative outcomes, and propose a thought experiment to help more fully understand the overlap between the structure of cognitive and creative aptitudes more broadly. Next, we delve into prospective research on cognitive aptitudes and STEM outcomes, including creativity. This includes thinking about a multitude of factors that go into the development of STEM creative expertise. Then, we consider factors in the developmental histories of top STEM graduate students and creative scientists by reviewing research taking a retrospective approach, and consider how these two approaches inform one another (e.g., Simonton, 2000, 2017; Feist, 2006). We discuss limitations and possible future directions and then consider social and cultural contexts that might fruitfully facilitate the talent development of creative scientists. Finally, we conclude the paper with some policy recommendations to enhance scientific creativity through talent development.

## If You Are Creative, Are You Also Intellectually Talented?

One of the oldest discussions regarding the measurement of aptitudes is the creativity and intelligence one (Barron, 1969; Sternberg and O'Hara, 1999; Kell et al., 2013; Plucker et al., 2020). The fields of cognitive abilities or aptitudes research and creativity or creative aptitudes research diverged many years ago. The cognitive aptitudes research community largely has agreed, at least to date, upon a hierarchical model of abilities (e.g., Carroll, 1993). The creativity community still remains somewhat fragmented regarding the measures which indicate what it means to be creative, though there is definitional agreement on the two components of originality/novelty and usefulness/meaningfulness (e.g., Kim, 2005; Plucker et al., 2020). In more recent years, however, there has been a resurgence of studies that have started to reconsider the relationship between cognitive aptitudes and creativity. Creativity researchers Nusbaum and Silvia (2011), for example, asked the question “Are intelligence and creativity really so different?” Thus, researchers from both sides of the aisle are beginning to take the possible construct(s) measurement overlap of the wide range of creativity and cognitive aptitude measures seriously, and perhaps a better understanding of the extent to which there is a jangle fallacy (Kelley, 1927) operating will become better known as more research is conducted. Understanding this overlap (or lack thereof) may have insights for understanding creative problem solving, better ways of identifying and developing talent, and also may inform the discussion around questions such as: what is the probability that you are highly creative given you are already quite smart vs. what is the probability that you are highly smart given you are already highly creative? Here, we examine recent summaries of the research on cognitive aptitudes and creativity. We are primarily measurement focused researchers most familiar with the evidence base surrounding cognitive aptitudes and use this as our conceptual starting point for consideration. We should note at the outset that all cognitive and creative aptitudes are *developed* and can be considered to be malleable through education or other means of talent development (e.g., Lohman, 1993, 2005; Subotnik et al., 2011; Ritchie and Tucker-Drob, 2018).

### Cognitive and Creative Aptitudes Measure Overlap

Plucker et al. (2020) helpfully summarized the empirical work on cognitive aptitude and creativity measures to date, pointing out much of the research on the relationship between creative and cognitive aptitude measures is largely dependent on the specific measures used, such as the Torrance Test of Creative Thinking (TTCT), a DT test, or other similar paper and pencil tests. The relationship in these types of studies has been argued to be low by some scholars (e.g., Barron and Harrington, 1981; Kim, 2005). However, a recent meta-analysis by Gerwig et al. (2021) on DT tests showed that the overall correlation between creativity and cognitive aptitude was *r* = 0.25 and that when “employing test-like assessments coupled with be-creative instructions and considering DT originality scores” the correlation could be higher (*r* = 0.31–0.37), concluding that the intelligence-DT correlation is quite robust. Scholars have also argued that true measures of the constructs and their overlap have been underestimated in many studies due to the focus on observable scores (Silvia, 2008). And in fact, studies that are able to assess the latent correlations between general cognitive and creative aptitudes estimate this at about *r* = 0.40 (Nusbaum and Silvia, 2011; Benedek et al., 2012; Karwowski et al., 2020; Weiss et al., 2021). These effect sizes are not quite as strong as the average intercorrelations between narrowly defined cognitive aptitudes as reported in meta-analyses (*r*s between 0.53 and 0.64; Bryan and Mayer, 2020) but well-within the range of correlations found between aptitude subtest scores (*r*s between 0.26 and 0.69; Lang et al., 2010). Therefore, current evidence indicates that creativity is related to, but not redundant with, more general forms of cognitive aptitudes. These estimates are also limited by the extent to which the cognitive measures used are in fact representative of the broader structure of aptitudes which is well-established (e.g., Carroll, 1993) and the extent to which the creativity measures used are representative of the broader structure of creative aptitudes, which is much less well-established (e.g., Plucker et al., 2020).

### Cognitive Aptitudes and Creative Achievement

#### Creative Achievement

The relationship between cognitive aptitudes and creative achievement test performance was recently meta-analyzed (Karwowski et al., 2021) and confirms an older meta-analysis (Kim, 2008) on the general population (*r* = 0.16 and *r* = 0.21 for unreliability corrected effect size). Specifically for science achievement, Karwowski et al. (2021) found that *r* = 0.19, and that this would be larger when corrected. Overall, this suggests moderate to large effect sizes for individual differences research (Gignac and Szodorai, 2016). Beaty et al. (2015) also show that these associations increase when the measurement error is accounted for in latent variable models. Karwowski et al. (2021) note that a core limitation of the literature reviewed in this area is that much of it has been conducted in the last decade primarily using one measure, the Creative Achievement Questionnaire, which is a self-report instrument (e.g., Carson et al., 2005), and additionally range restriction on the measures used may be an issue.

#### Long-Term Creative Achievement

Another core limitation that Karwowski et al. (2021) acknowledge is their meta-analysis excluded various populations and measures, including intellectually talented or gifted student samples. Much of the research to date illustrating a long-term link between early identified cognitive aptitudes and later creative achievement have been from the Study of Mathematically Precocious Youth (SMPY; Lubinski and Benbow, 2006) of which we will review more findings later. However, we will note here that SMPY has shown that early aptitudes are linked to rare, high-level creative achievements, such as obtaining patents, publications, and even university tenure (e.g., Wai et al., 2005; Park et al., 2007; Ferriman-Robertson et al., 2010).

### Threshold Hypothesis

Some scholars have hypothesized that cognitive aptitudes and creativity are positively related, but this relationship either fails to persist or becomes negative beyond a certain level. This notion is commonly referred to as the *threshold hypothesis* and has been used to explain why some anecdotal evidence suggests that highly creative individuals are not always cognitively talented (Andreasen, 2014). Others have pointed to Terman's famous study of gifted children as an indication that high cognitive aptitude does not guarantee creative eminence later in life (Feldman, 1984). The threshold is an alluring idea in that it supposes that most people are capable of being highly creative or achieving creative eminence given that there is no greater advantage to be gained beyond a certain level of cognitive talent, or perhaps that even more talent might at some point become detrimental. The exact point at which this advantage is thought to cease is uncertain but many scholars cite Guilford (1967) as initially proposing this nonlinear relationship. In some cases, the threshold hypothesis is framed as indicating that cognitive aptitudes are a necessary but not sufficient condition for creativity (Karwowski et al., 2016). Here, studies have used necessary condition analysis to illustrate that creative achievement later in life is unlikely for those with below average childhood cognitive reasoning but that high childhood aptitude does not always lead to greater achievement (Karwowski et al., 2017). As one possible interpretation, high cognitive talent may be important for access, in that doing well-enough academically to get into a top flight graduate program is important for the opportunity to be creative in STEM fields. An alternative version of this is the interference hypothesis, where some expect that creativity is obstructed by high levels of cognitive aptitude (Plucker et al., 2020). Others have suggested that this threshold effect might be moderated by personality such that a threshold is only apparent for individuals who are low in openness to experience (Harris et al., 2019). Moreover, Corazza and Lubart (2021) include time and space into their theoretical model to help explain the varying relationships between cognitive aptitude and creativity.

Despite continued interest in the threshold hypothesis, our reading of the literature is that there is little empirical evidence in support of this phenomenon to date, even though there have been multiple studies across the decades. Past narrative reviews have reported mixed findings (e.g., Karwowski and Gralewski, 2013) but several of the studies purporting to find support for a threshold were based on relatively small samples (e.g., Jauk et al., 2013; Welter et al., 2016). Even when a threshold is observed, there does not seem to be a consistent point at which it occurs (Shi et al., 2017). In contrast, several larger-scale studies and reviews have failed to support the existence of a threshold effect (Kim, 2005; Preckel et al., 2006; Weiss et al., 2020). Furthermore, others have found positive, linear relationships with creativity even within select cohorts of exceptionally talented individuals (Ferriman-Robertson et al., 2010). And ultimately, even Guilford, who originated or at least popularized the idea himself, was unable to find support for a threshold effect, as noted by Weiss et al. (2020). Based on the present body of research, there appears to be little consistent evidence for the threshold hypothesis between cognitive aptitudes and creativity, though further research in the area may illuminate new directions for this subarea of research to unfold.

### A Thought Experiment to Help Understand the True Overlap Between Measurement Structures: Utilizing the Multi-Trait, Multi-Method Matrix, and Bifactor Modeling

Despite the resurgence of interest in studying the measurement overlap between cognitive aptitude measures and creativity measures that have been developed to date, most of the meta-analytic studies and other individual controlled studies of specific measures have been limited to understanding the overlap and non-overlap between only the specific measures used and whatever construct(s) those measures tap. To date, however, there has not yet been a major factor analytic study similar to that done by Carroll (1993) which also includes all of the creativity measures as well. The structure of creativity is not agreed upon (e.g., Plucker et al., 2020), however, the incremental validity of the structure of creativity over and above the largely established hierarchical model of aptitudes (Carroll, 1993) is nowhere near understood (for discussion see Plucker and Esping, 2015; Gerwig et al., 2021). Relative to cognitive aptitudes, which are generally measured using standardized tests, there has also been little consensus regarding how creative aptitudes should be assessed (Said-Metwaly et al., 2017). As a result, cognitive aptitude testing is widely recommended for identifying talent in educational and occupational settings (Schmidt and Hunter, 2004). Therefore, we propose as a kind of thought experiment for how researchers can establish a model of creative aptitudes which are distinct from cognitive aptitudes as evidenced by discriminant validity. Not only would this help create a theoretical structure of creative aptitudes, but this would also potentially help increase the breadth of criteria used to identify creative and scientific potential and improve access to educational and training opportunities.

**Figure 1:**
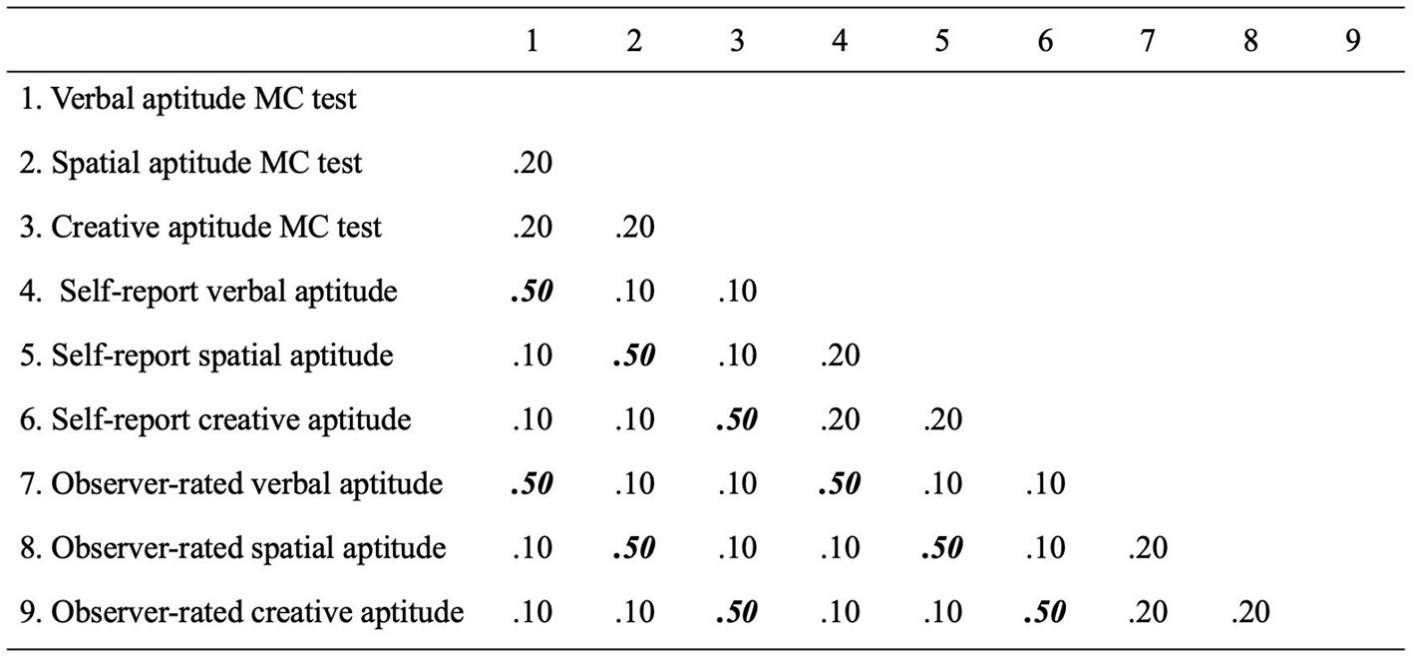
Example multi-trait multi-method (MTMM) matrix with measures of cognitive and creative aptitudes. Correlations in bold and italics represent convergent validity estimates between different methods of measuring a shared construct (0.50). Divergent validity can be determined by comparing the same trait—different method correlations with the correlations among different trait—different method (all shown as 0.10) and different trait—same method combinations (all shown as 0.20).

One method for testing the discriminant validity of creative from cognitive aptitudes is the mult-trait, multi-method matrix (MTMM; Campbell and Fiske, 1959). The MTMM allows researchers to quantify the extent to which the correlation between two constructs can be explained by shared method factors (e.g., self-report or ability test formats) or shared variance between the constructs themselves (across different measurement methods). In a hypothetical MTMM study, we would collect numerous measures of cognitive and creative talents using a variety of methods such as a battery of multiple-choice aptitude tests, self-report ratings, or observer ratings. This design would yield a correlation matrix of constructs fully crossed with measurement methods (see Figure 1).

This could then be used to determine the relative contributions of method and construct factors to the observed correlations between cognitive and creative talents. First, this matrix could be used to determine how strongly different types of measures converge with other measures of the same construct (correlations shown as 0.50 in Figure 1). This is generally considered evidence for convergent validity. More importantly, the MTMM could be used to determine discriminant validity by comparing correlations when using different methods to those obtained when using the same method (correlations shown as 0.20 in Figure 1). For example, do different methods of measuring creativity correlate more strongly than measures of different constructs (cognitive and creative talent) using a shared method? This would help identify whether the covariance between these constructs represent a true relationship between creativity and cognitive aptitudes or are mostly a function of using similar measurement methods.

**Figure 2:**
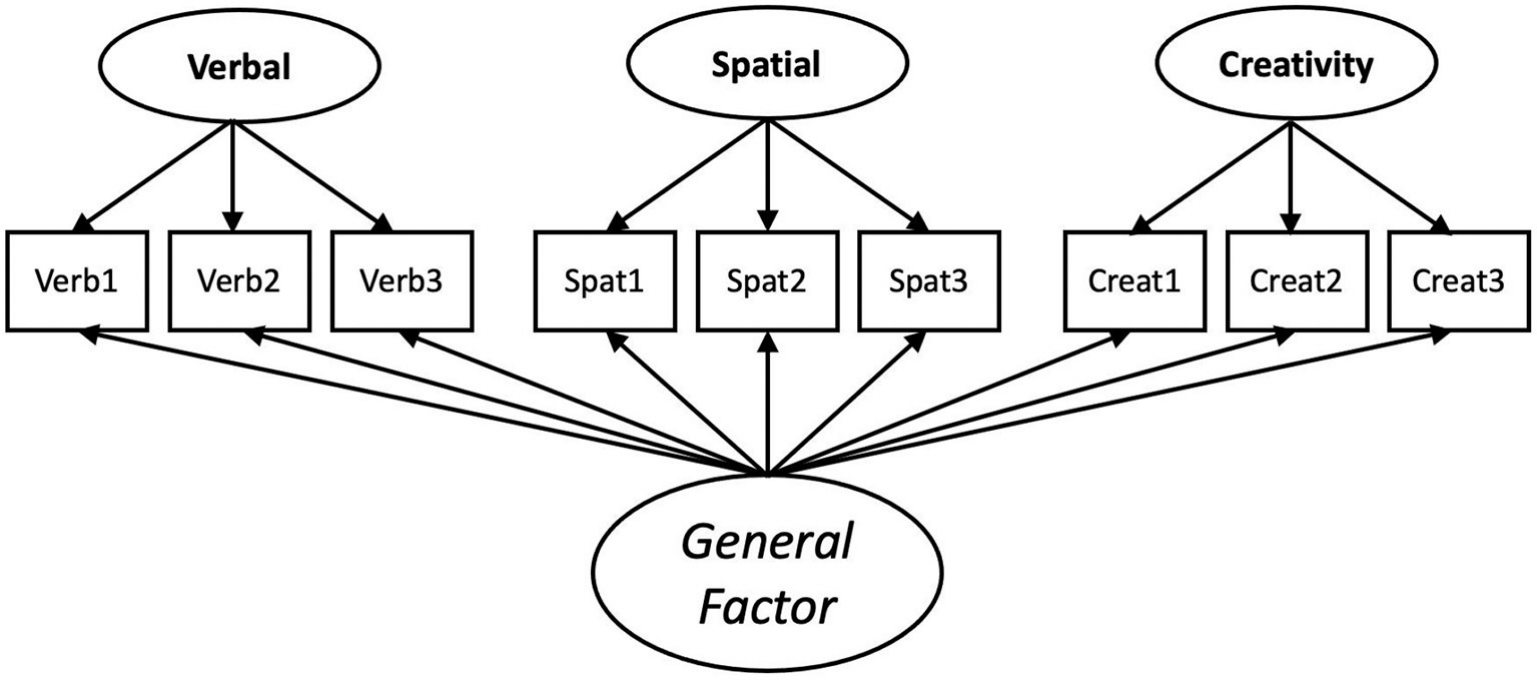
Example bifactor model using measures of verbal aptitude, spatial aptitude, and divergent thinking and a general factor.

Another method for determining the overlap between different constructs is bifactor modeling. In a bifactor model, each individual item or scale is modeled as an indicator of a latent general factor and a latent specific group factor. This structure provides the ability to determine the extent to which test score variance is explained by a general factor or by more narrowly defined group factors (see Figure 2). For cognitive tests, bifactor models have been found to provide better overall fit compared to hierarchical models (e.g., Cucina and Byle, 2017). More importantly, bifactor models have been used to demonstrate the unique effects of narrow aptitude measures beyond general reasoning, such as emotional intelligence (MacCann et al., 2014). In our example, the factor loadings for the DT factor indicates the degree to which the three tests can reliably measure DT independent of general reasoning. These factor loadings can also be compared to loadings onto a general factor. Although researchers have yet to use this approach to test for the discriminant validity of creative ability tests, some recent works have used bifactor modeling to illustrate the effects of second-level cognitive abilities, like verbal fluency (Silvia et al., 2013), or a general factor of executive attention (Frith et al., 2021) on DT task performance.

Despite the usefulness of the MTMM or bifactor modeling, few studies have used these methods to test the discriminant validity of creativity measures relative to cognitive aptitude measures at all, let alone a broad array of measure types. Older works used a MTMM design for analyzing tests of creativity (Bachelor, 1989) or DT (Hocevar and Michael, 1979), but this method has not been fully utilized to test for the discriminant validity between measures of creativity and cognitive talents. This is an important oversight given that measures of creativity have sometimes been found to correlate more strongly with cognitive aptitude measures compared to other creativity tests (Wallach and Kagan, 1965) and the size of the correlation between these measures varies between different types of creativity measures (Kim, 2008). Moreover, researchers now have more rigorous tools, such as structural equation modeling, for analyzing MTMM data (Eid et al., 2008, 2016) and estimating bifactor models (Reise, 2012). We believe that these study designs are useful for identifying a broader range of creative aptitudes which can be reliably measured and used to identify creative talent beyond what is measured in standardized cognitive tests. These approaches have proven useful for advancing research on cognitive aptitudes but have yet to be widely applied in creativity research. Thus, we hope our thought experiment may serve as a useful guide for future research in this area.

## Prospective Research on Cognitive Aptitudes and STEM Outcomes, Including Creativity

As noted earlier in our discussion regarding the threshold hypothesis, a greater degree of developed aptitude appears to be beneficial in the sense of improving the likelihood of a wide range of educational, occupational, and life outcomes, not just limited to but also including STEM (e.g., Lubinski and Benbow, 2006; Park et al., 2007; Brown et al., 2021). This means that focusing on prospective samples of intellectually talented or gifted students who have had their talents largely developed and have also been followed up over the decades can provide some insights into the attributes, educational factors, and other contextual variables that can contribute to the development of creative scientific or STEM expertise. This review is not meant to be comprehensive of all prospective longitudinal studies relevant to this topic (e.g., see Subotnik and Steiner, 1992; Feist and Barron, 2003 as a starting point for other relevant literature), but focused primarily on SMPY.

### Prospective Research and Outcomes

The Study of Mathematically Precocious Youth (Lubinski and Benbow, 2006, 2020) is a planned 50-year longitudinal lifespan developmental study originally founded in 1971 of over 5,000 talented youths across five cohorts. Four of the cohorts were identified in the 7th grade as part of a talent search process starting in the 1970s using the SAT, which was designed for high school (HS) students, as a way to provide sufficient measurement headroom to distinguish the full range of aptitudes among exceptional performers. Currently at talent search centers across the US such as Johns Hopkins University and Northwestern University, students can take the SAT or ACT in the 7th grade. As part of the emphasis of SMPY has been on understanding the developmental antecedents to STEM accomplishment and creative achievement (Lubinski and Benbow, 2006), a fifth cohort was identified as a sample of highly select US STEM graduate students to examine whether findings from SMPY generalized to extraordinary STEM talent (which will be further discussed later in the retrospective research section). The SMPY study is uniquely positioned to examine prospective research on cognitive aptitudes and STEM outcomes and thus is emphasized here. Another unique sample of over 400,000 students using the talent search population across roughly the last two decades on students who took the ACT will also be reviewed (Wai and Allen, 2019). Finally, population representative samples from multiple longitudinal studies will be highlighted to illustrate that findings from the select talent search samples also reasonably generalize to a random sample of the intellectually talented or gifted population.

### SMPY: Factors That Go Into the Development of Creative Expertise in STEM

As the SMPY study deliberately used the SAT as the measure of cognitive aptitudes in addition to some spatial reasoning measures for some students, the core specific aptitudes examined and selected for are from the Radex of Carroll's (1993) hierarchical model, which draws from general reasoning as well as math, verbal, and spatial components (Lubinski and Benbow, 2000). Thus, this study examines what happens when you identify students as talented using cognitive aptitude measures and seeing to what extent such students end up being creative in STEM or across a wide range of other domains. Lubinski and Benbow (2006, 2020) summarize some of the variables they've examined to help identify STEM talent and also provide such talent with appropriate STEM focused educational interventions and stimulation in order to develop their talents into eventual STEM educational, occupational, and creative expertise. They note that math and spatial talents as well as investigative and theoretical interests form an aptitude complex (Ackerman, 2003) that seems to be associated with STEM achievement. Building upon these aptitudes with special educational opportunities, or enhanced educational dosage, both inside and outside of school, in addition to personal commitment and motivation of students toward putting in the deliberate practice needed to rise to the top of a field can ultimately improve the likelihood of someone eventually achieving STEM creative expertise.

### Math and Spatial Aptitudes for STEM Expertise

Specifically, math and spatial aptitude have been determined by SMPY and numerous other longitudinal studies to be linked with STEM and other outcomes. An analysis linking data reviewed from Super and Bachrach (1957), a longitudinal study known as Project Talent which was a random sample of the US population identified in the 1960s and followed up over many years, and SMPY longitudinal findings using cohorts tested on spatial reasoning, have illustrated that spatial talents in particular are useful for STEM educational and occupational pursuits across the last half century or more (Wai et al., 2009). Math reasoning was also important to STEM outcomes in addition to spatial reasoning, illustrating that relatively higher developed math and spatial aptitudes compared to verbal aptitudes fit the average profile of many STEM disciplines. Kell et al. (2013) illustrated that spatial reasoning mattered for many creative STEM and technical innovation outcomes, such as STEM publications and patents. This aligned with other research from SMPY which showed that though both early identified math and verbal aptitudes combined predicted level of achievement, it was the “tilt pattern” of aptitudes, or the profile of specific talents, in particular math aptitude higher than verbal aptitude, that mattered for later STEM and creative outcomes many years later in life (Park et al., 2007). As math reasoning is typically already used in many gifted education selection procedures due to being included in many K-12 and other standardized tests used in higher education and beyond, Lakin and Wai (2020) estimated, using three nationally representative samples that spanned the last 60 years, that roughly 2–3 million students in US K-12 education have higher spatial than math or verbal talents and are thus neglected in schools in terms of gifted selection and appropriate educational curriculum suited to their profile of strengths (Wai and Lakin, 2020). We will return to this point later about how leveraging untapped pools of talent such as students with spatial reasoning strengths could significantly enhance the development and broadening of the pool of STEM creative scientists and innovators, especially from disadvantaged backgrounds.

### Broader Aptitude Complexes for STEM Expertise

A series of longitudinal studies from SMPY has also illustrated the role that broader aptitude complexes can play as developmental markers for eventual STEM achievement and creative pursuits (Achter et al., 1999; Wai et al., 2005; Bernstein et al., 2019). The most recent study by Bernstein et al. (2019) showed how developed math/scientific and verbal/humanistic aptitude and preference constellations in talented students identified at age 13 (Achter et al., 1999) were associated with educational outcomes at age 23 (Wai et al., 2005) and illustrated that by age 35 these constellations continued to be associated with different forms of eminence. In particular, the math/scientific constellation predicted outcomes by age 35 in STEM areas, including attaining professorships, grants, patents, and being an executive of a STEM Fortune 500 company. The math/science aptitude complex was composed largely of relatively higher math aptitude than verbal aptitude and also relatively higher theoretical and economic interests as part of the Study of Values measure of preferences.

### The Role of STEM Educational Opportunities for Optimal Development

Early identified math and spatial aptitude, including broader constellations of aptitude complexes, have thus been useful in forecasting later STEM achievement and creativity. Here, we discuss how talent development through educational or other intellectually stimulating activities in a wider sense can help fully develop the expertise of STEM creative achievers.

The first study we review is based on data from SMPY, which introduced the concept of educational dosage, adapted from the use of the term dosage in health contexts (Wai et al., 2010). Just as in health what likely matters is the right mix of exercise or wellness opportunities, the idea behind educational dosage is that each student gets the right mix of educational opportunities. This conceptualization is along the lines of the idea that each student should at least learn something new each day (Stanley, 2000) and that consistent, and perhaps a wide variety, of learning opportunities should cumulatively add up over time in the development of expertise broadly. This does not preclude the importance of rigorous research designs to help disentangle the causal impact of educational interventions apart from selection bias (e.g., Schlotter et al., 2011; Singer, 2019), and most certainly does not mean that some interventions are not more effective than others. However, by conceptualizing education broadly and perhaps some types of educational opportunities as somewhat functionally interchangeable, this allows students to take advantage of stimulating intellectual opportunities that they have access to at a given point in time and geographic place in their development and are interested in pursuing to help enhance their eventual talent development. Based on this conceptualization, Wai et al. (2010) quantified STEM educational dosage as the number of different STEM educational opportunities talented students had in their pre-college years and compared the higher and lower educational dosage groups in terms of long-term STEM outcomes. Even after accounting for math aptitude to some degree, the group with higher STEM educational dosage ended up having a higher rate of earning STEM doctorates, publications, patents, occupations and even university tenure.

The second study we review here is also based on a sample of talented students who took part in the 7th grade talent search in the US from 1996 to 2017, and in particular took the ACT (Wai and Allen, 2019). This study linked 7th grade data to scores of the same students who also took the ACT in the 11th/12th grades as well as data on sociodemographics, interests, HS characteristics, HS GPA and coursework, and extracurricular activities. Then, in this sample of 482,418 students, these predictors were used to examine which factors had the highest associations with academic growth in ACT scores between the 7th grade to the 11th/12th grade from 1996 to 2017. Broadly, academic growth was associated with greater participation in advanced learning opportunities such as advanced AP, accelerated, honors courses, and elective HS courses, but in particular STEM elective HS courses.

## Retrospective Research on STEM Graduate Students and Creative Scientists

Another approach to the study of creativity in science is the retrospective approach, or a look back into developmental histories, typically taken historically in much of the research on expertise across various fields (e.g., Roe, 1953a,b; Simonton, 1994, 1998, 2017; Root-Bernstein and Pawelec, 2016; Wai and Rindermann, 2017). For example, Grosul and Feist (2014), based on their examination of academic physical, biological, and social scientists from major research universities, uncovered that personality traits may be related to scientific creativity. Though this type of research design can be considered selection on the dependent variable which makes understanding the true nature of causal factors very challenging to untangle, there is still insight to be gained by descriptively or qualitatively studying extraordinary experts to better understand the attributes of those who made it to the very top of the profession (Gerring, 2012). Here, we briefly review some of the retrospective research on STEM graduate students from SMPY and then also discuss attributes and experiences of individuals who made it to the very top of STEM creative achievement or innovation.

### Findings From Top STEM Graduate Students

Some of the papers reviewed earlier combined a prospective and retrospective approach to better understand talent development of gifted students who ended up many years later in STEM fields and STEM graduate students who were very likely talented at an earlier age when looking back in their developmental histories. In particular, the findings on educational dosage from Wai et al. (2010) and the math/science aptitude preference constellations (Bernstein et al., 2019) were also replicated within the top STEM graduate student sample from SMPY (Lubinski and Benbow, 2006).

The most recent review and update of the accomplishments of the top STEM graduate student sample as identified by SMPY originally in 1992 is by McCabe et al. (2020). In this study, the authors were specifically interested in which of those students they followed over the last 25 years ended up as a STEM leader: defined as STEM full professors at top research universities, STEM leaders in government, and STEM CEOs. They used a non-STEM leader group for comparison with a specific focus on gender. Overall, the study found that individual differences assessed early in graduate school were associated with becoming a STEM leader, not just on cognitive aptitudes but also developed interests, values, and personality. Science, technology, engineering, and mathematics leaders tended to be highly focused on work and worked more hours than non-leaders. Men tended to be more interested in STEM and were more career focused. Women had a wider range of interests including activities outside of work and career.

### Findings on the Educational Backgrounds and Training Environments of Top Creative Scientists

A recent study using data from the TIME 100 going back to its inception also examined data from scientists and thinkers who were selected as one of the 100 most influential people in the US during that year (Wai et al., 2019). Overall, roughly 60–80% of the scientists/thinkers identified across the years in the TIME 100 attended a set of highly select schools that often required very high average test scores indicating cognitive aptitudes roughly in the top 1% (similar to the level of the SMPY sample), suggesting that at least in this unique highly selected sample, creative scientists and thinkers tended to have high aptitude with also the majority having attended highly selective institutions and perhaps benefitting from the educational, peer, or other network benefits of such schools. One core limitation of this study was that thinkers could not be separated from the scientists. These results confirm, at least in part, some of the early findings from others many decades ago (e.g., Roe, 1953a,b).

A different type of analysis sought to examine the educational backgrounds of top creative scientists by seeking to uncover which schools had produced, throughout history, the most Nobel prize winners, per capita (Hsu and Wai, 2015; Clynes, 2016; Wai, 2020). The driving idea was to isolate the top producing undergraduate institutions to understand not only the early training grounds that encouraged highly accomplished STEM creatives but also consider how students were selected for entry into those schools in the first place, for example, in part for their high cognitive and non-cognitive aptitudes. Overall, the top 10 schools for most scientific prizewinners were: 1. École Normale Supérieure (France), 2. California Institute of Technology (US), 3. Harvard University (US), 4. Swarthmore College (US), 5. Cambridge University (UK), 6. École Polytechnique (France), 7. Massachusetts Institute of Technology (US), 8. Columbia University (US), 9. Amherst College (US), 10. University of Chicago (US). As this analysis sought to correct for winners per capita by estimating the population across history that had graduated from each institution, naturally, longer standing institutions such as Harvard had an advantage. At the same time, smaller institutions rose to the top of the rankings, in particular the California Institute of Technology in the US and École Normale Supérieure in France. These two schools, in particular, are typically highly selective on aptitudes and also very likely are STEM focused training grounds for highly gifted, achieving, and motivated STEM interested students. In addition, a broader analysis using similar methods but also looking at the US National Academies of Sciences, Engineering, and Medicine, as well as Turing Prize winners (computer science) and Fields Medalists (mathematics) also turned up rather similar results (Hsu and Wai, 2015). Thus, looking at the ultimate outcomes achieved by graduates of universities may be one important way to examine to what extent these selection and training environments might improve ultimate STEM creative achievement like winning a Nobel Prize. And perhaps understanding why such training environments have produced such outstanding creative scientists may be useful in uncovering what types and intensity of educational dosage or quality of peer environments are most effective at the undergraduate level or above for STEM talent development, potentially informing interventions and training in other contexts.

## Limitations and Possible Future Directions

The selected review of the literature in this paper on some likely antecedents to STEM creative expertise provides an important, though of course cognitive aptitude focused examination of what developed factors go into creative expertise along with numerous other educational and contextual factors discussed (and many others not reviewed). Researchers more focused on creativity measures and constructs might highlight how there is an important aspect of non-overlap between creativity and cognitive aptitude measures (e.g., Plucker et al., 2020) and also note that longitudinal studies focused on creativity rather than cognitive aptitudes come to different conclusions favoring creative aptitudes over cognitive ones (e.g., Plucker, 1999; Runco, 1999). We note that our proposal to better understand the measurement overlap and non-overlap between cognitive aptitude and creativity measures through our thought experiment utilizing an MTMM or bifactor modeling approach would significantly enhance our understanding of how the different fields overlap (or are distinct) in their measurement. Also, more consideration of how large-scale longitudinal studies that focus on developed cognitive or creative aptitudes early in life may help better understand measurement overlap and non-overlap in terms of ultimate prediction of a wide variety of life outcomes, including STEM creative achievement. Finally, we note that STEM is a broad and quite diverse set of individual and unique fields and that because these domains have evolved over time more contemporary studies may be more useful to addressing questions about the development of gifted students who may build science, technology, engineering, or mathematical expertise today and in the future.

## Social and Cultural Contexts to Facilitate Talent Development of Creative Scientists

The research reviewed up to this point has largely focused on prospective and retrospective studies that examined developed aptitudes, preferences, and educational dosage in relation to STEM achievement at various levels. These findings have been replicated in history across different time points in various datasets thus these findings are robust across time to some degree. At the same time, social and cultural contexts, whether in K-12 or higher education, broader societal circumstances such as the COVID-19 pandemic, and the scientific culture and incentive structure that scientists must pass through to make long-term contributions to the scientific record are all shaped by these social, cultural, and institutional factors, including incentive structures. These factors vary as a function of the specific stage of talent development, country, culture, institution, and various other dimensions, but here we briefly discuss some aspects along the developmental timeline that may matter for the facilitation of STEM talent to improve the likelihood of enhanced creative STEM expertise and ultimately novel scientific contribution and advancement.

As the review of the SMPY longitudinal studies illustrates, students who have the opportunity to more fully develop their talents over time are more likely to innovate in a wide range of areas, including in STEM (e.g., Lubinski and Benbow, 2006, 2020). This suggests that talent development requires social and cultural contexts in the form of educational opportunities and learning environments that can help facilitate that talent development throughout the K-12 years (Stanley, 2000; Wai et al., 2010) and also into college and graduate school where talent development of top scientists largely happens in the highest ranking and most competitive programs with unique selection and training models (e.g., Clynes, 2016; Wai, 2020). One important emphasis in this context should be on disadvantaged but talented students, especially those from marginalized and underrepresented minority backgrounds, who very likely are falling through the cracks due to a lack of attention to their educational resource and other needs and numerous hurdles that they face, including societal and structural factors, that likely accumulate over development (Wai and Worrell, 2020). For example, talented but low-income students likely face many headwinds, whereas talented but more advantaged students have the advantage of many tailwinds, thus deepening the divide between students who have the aptitudes and other potential for ultimately contributing to STEM areas and innovations but simply do not have the *opportunity* to develop their talents to the fullest (e.g., Lakin and Wai, 2020; Wai and Worrell, 2020). Parents will almost always support the needs of their own children, which suggests that talented students without resource rich parents (whether money, time, education, family support, or otherwise) largely depend upon educational opportunities that are provided in school. We discuss some policy recommendations to improve talent development in the next section, but stress here that focusing on helping develop talents of the disadvantaged is important as some students, due to poverty or other headwinds, may not even have the opportunity to develop to be gifted (e.g., Hair et al., 2015).

Focusing on helping talented but marginalized students with early developed aptitude constellations for STEM domains (e.g., Bernstein et al., 2019) throughout K-12 might then improve their representation in selective STEM training environments at both the college and then graduate school level (e.g., McCabe et al., 2020). Undergraduate and graduate training at these top STEM institutions (e.g., understanding the training environments of École Normale Supérieure or the California Institute of Technology; Clynes, 2016) may serve as important socialization that provides a foundation for the eventual development of STEM expertise and the ability to make a truly novel and useful scientific advance.

Beyond graduate training there needs to be more attention paid to the incentive structure in science. For example, the sociologist Merton (1968, 1973) highlighted the importance of understanding how the reward structure in science works (de Solla Price, 1986; Jones et al., 2014), stressing that winner take all markets where those who have get even more, a Matthew effect, often widens inequality in scientific outcomes ranging from who obtains tenure track jobs to who gets funding such that a Nobel Prize winning achievement can even take place (Stephan, 2010; Anderson and Geist, 2017; Bol et al., 2018). By recognizing how the structure currently functions, perhaps we might be able to then improve that structure to improve the likelihood that scientists and their incentives are focused on the appropriate goals that can lead to true scientific advance, which may not always be aligned with the reward structure of science (for example, what is arguably the negative impact of grant culture on scientific advance in some contexts, Lilienfeld, 2017). Overcoming structural barriers may be especially important when designing policies to support women in science (Ceci et al., 2014) in particular when it comes to childcare and household division of labor, which may have exacerbated inequalities during the pandemic (e.g., Zamarro and Prados, 2021). This gender gap in STEM becomes even more apparent when focusing only on extremely productive, star performers where men have been found to increasingly outnumber women within the top 10% of publishing academic researchers (Aguinis et al., 2018).

The academic incentive structure in many fields increasingly remains rather siloed in terms of obtaining and progressing in tenure track positions and securing grant funding, and ultimately solutions to improve the incentive and cultural environments for truly interdisciplinary scholars to be able to have academic careers is incredibly important (Lyall et al., 2011; Lyall, 2019). Of course, scientific advance is happening in many different entrepreneurial, business, and other contexts, such as research for the government and various private companies, and learning from all these contexts would be useful to understand where scientists can most fruitfully make novel contributions to scientific understanding and to society.

## Policy Recommendations to Enhance Scientific Creativity Through Talent Development

Research at the global level indicates that students who have developed very high cognitive aptitudes as measured by international comparison tests (Angrist et al., 2021) and the developed knowledge capital they measure are important to scientific innovation (e.g., Rindermann and Thompson, 2011; Hanushek and Woessmann, 2015). Heckman (2000), in an article titled “Policies to foster human capital,” noted that early human capital or educational investment in highly talented students would have a very high long-run payoff in economic aspects and innovation, and when linked with the SMPY findings reviewed in this paper illustrating how fully developed talented students contribute significantly to innovation in a wide variety of forms (e.g., Lubinski and Benbow, 2006; Kell et al., 2013; McCabe et al., 2020), this suggests that investing in talented students, especially students from marginalized backgrounds (e.g., Wai and Worrell, 2020), could greatly improve equity and access to the STEM talent development pipeline and ultimate STEM creative achievement. Of course, in particular for talent from marginalized and underrepresented groups there needs to be policies in place to support and retain such faculty, including women in STEM (e.g., Ceci et al., 2014; Zamarro and Prados, 2021).

Given that the majority of standardized tests throughout K-12 and even in higher education admissions include math and verbal aptitude measures but fail to include spatial measures means that we are missing millions of spatially talented kids, many from disadvantaged and low income backgrounds, who would benefit from the opportunity to develop their talents through educational opportunities and curriculum suited to their spatial strengths (Lakin and Wai, 2020). In addition, spatial reasoning has been linked to a wide variety of STEM achievement and innovation (Kell et al., 2013) and can be developed through training (e.g., Uttal et al., 2013; Sorby et al., 2018), similar to all abilities (Lohman, 1993, 2005; Subotnik et al., 2011; Ritchie and Tucker-Drob, 2018). Math and verbal reasoning are important for performance in schools, and universal screening even using such measures have been shown to improve the identification of disadvantaged and underrepresented minority talent (Card and Giuliano, 2016). Thus, our proposal to expand measures to capture spatial reasoning should not be viewed as saying that tests are not useful at present. They can be useful if utilized in an appropriate way, such as using them along with other converging indicators.

Educational acceleration, or moving students through the regular curriculum at rates faster than typical (e.g., Assouline et al., 2015), has been shown to be a useful class of interventions to improve STEM achievement and productivity (for example, grade skipping: Park et al., 2013). Universal screening on multiple STEM aptitudes reviewed here (Card and Giuliano, 2016), perhaps calibrated to local opportunities to learn, could then help match students to STEM educational dosage over many years of sustained training that could significantly enhance the development of STEM creative expertise. By focusing on broadly identifying and then fully developing underappreciated talent throughout K-12 education and development not only in the US but all across the world (e.g., Rise program, https://www.risefortheworld.org/; World Science Scholars, https://www.worldsciencefestival.com/education/world-science-scholars/), this can benefit all of us through scientific advances. Such global talent searches are also examples of how scientific creative achievement might also be celebrated culturally. More consideration of how STEM talent is truly global and policies to ensure talent is supported no matter where in the world it is being developed is also important [e.g., West, 2011; National Foundation for American Policy (NFAP), 2017].

## Author Contributions

JW and MB conceptualized and wrote the manuscript. Both authors contributed to the article and approved the submitted version.

## Conflict of Interest

The authors declare that the research was conducted in the absence of any commercial or financial relationships that could be construed as a potential conflict of interest.

## Publisher's Note

All claims expressed in this article are solely those of the authors and do not necessarily represent those of their affiliated organizations, or those of the publisher, the editors and the reviewers. Any product that may be evaluated in this article, or claim that may be made by its manufacturer, is not guaranteed or endorsed by the publisher.
